# 2,3-Dihydro-1*H*-cyclo­penta­[*b*]naph­tha­len-1-ol

**DOI:** 10.1107/S1600536812005181

**Published:** 2012-02-10

**Authors:** Ísmail Çelik, Mehmet Akkurt, Ahmet Tutar, Ramazan Erenler, Santiago García-Granda

**Affiliations:** a Department of Physics, Faculty of Arts and Sciences, Cumhuriyet University, 58140 Sivas, Turkey; b Department of Physics, Faculty of Arts and Sciences, Erciyes University, 38039 Kayseri, Turkey; c Sakarya University, Faculty of Arts and Sciences, Department of Chemistry, 54187 Adapazarı, Turkey; d Gaziosmanpaşa University, Faculty of Arts and Sciences, Department of Chemistry, 60240 Tokat, Turkey; e Departamento Química Física y Analítica, Facultad de Química, Universidad Oviedo, C/ Julián Clavería, 8, 33006 Oviedo (Asturias), Spain

## Abstract

In the title compound, C_13_H_12_O, the cyclo­pentene ring fused with the naphthalene group adopts an envelope conformation. The cyclo­pentene torsion angle, with the fusion bond at the centre, has a magnitude of 1.16 (16)°. In the crystal, neigh­bouring mol­ecules are connected through O—H⋯O hydrogen bonds into an *R*_4_^4^(8) ring motif. The crystal packing also features weak π–π stacking inter­actions [centroid–centroid distance = 3.8981 (8) Å] and C—H⋯π inter­actions.

## Related literature

For the synthesis of the title compound, see: Carpino & Lin (1990 ▶). For the crystal structure of a similar compound, see: Çelik *et al.* (2009 ▶). For puckering parameters, see: Cremer & Pople (1975 ▶). For graph-set analysis, see: Bernstein *et al.* (1995 ▶). For bond-length data, see: Allen *et al.* (1987 ▶).
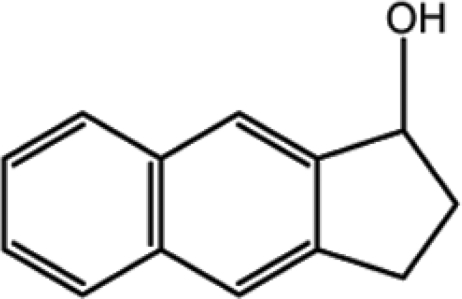


## Experimental

### 

#### Crystal data

C_13_H_12_O
*M* *_r_* = 184.23
Tetragonal, 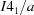

*a* = 25.3105 (4) Å
*c* = 6.0995 (2) Å
*V* = 3907.47 (18) Å^3^
*Z* = 16
Cu *K*α radiation
μ = 0.61 mm^−1^
*T* = 299 K
0.46 × 0.17 × 0.11 mm

#### Data collection

Agilent Xcalibur Ruby Gemini diffractometer
Absorption correction: multi-scan (*CrysAlis PRO*; Agilent, 2011 ▶) *T*_min_ = 0.884, *T*_max_ = 0.936
5702 measured reflections
1791 independent reflections
1580 reflections with *I* > 2σ(*I*)
*R*_int_ = 0.026

#### Refinement

*R*[*F* ^2^ > 2σ(*F* ^2^)] = 0.044
*wR*(*F* ^2^) = 0.126
*S* = 1.04
1791 reflections
131 parameters
H atoms treated by a mixture of independent and constrained refinement
Δρ_max_ = 0.10 e Å^−3^
Δρ_min_ = −0.17 e Å^−3^

### 

Data collection: *CrysAlis PRO* (Agilent, 2011 ▶); cell refinement: *CrysAlis PRO*; data reduction: *CrysAlis PRO*; program(s) used to solve structure: *SHELXS97* (Sheldrick, 2008 ▶); program(s) used to refine structure: *SHELXL97* (Sheldrick, 2008 ▶); molecular graphics: *ORTEP-3 for Windows* (Farrugia, 1997 ▶); software used to prepare material for publication: *WinGX* (Farrugia, 1999 ▶) and *PLATON* (Spek, 2009 ▶).

## Supplementary Material

Crystal structure: contains datablock(s) global, I. DOI: 10.1107/S1600536812005181/qm2052sup1.cif

Structure factors: contains datablock(s) I. DOI: 10.1107/S1600536812005181/qm2052Isup2.hkl

Supplementary material file. DOI: 10.1107/S1600536812005181/qm2052Isup3.cml

Additional supplementary materials:  crystallographic information; 3D view; checkCIF report

## Figures and Tables

**Table 1:** Hydrogen-bond geometry (Å, °) *Cg*2 is a centroid of the C1–C6 benzene ring.

*D*—H⋯*A*	*D*—H	H⋯*A*	*D*⋯*A*	*D*—H⋯*A*
O1—H1O⋯O1^i^	0.93 (2)	1.78 (2)	2.7113 (15)	174.4 (18)
C3—H3⋯*Cg*2^ii^	0.93	2.71	3.633 (2)	171
C11—H11*A*⋯*Cg*2^iii^	0.97	2.89	3.706 (2)	142

Symmetry codes: (i) 
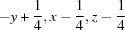
; (ii) 
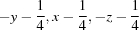
; (iii) 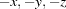.

**Fig. 1.**
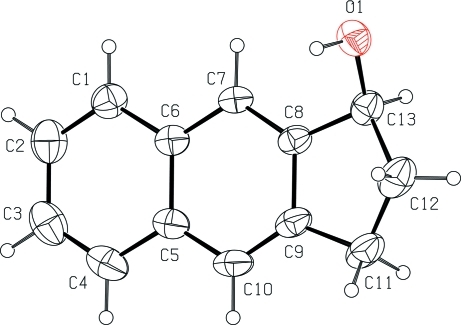
The title molecule with the atom numbering scheme. Displacement ellipsoids for non-H atoms are drawn at the 30% probability level.

**Fig. 2.**
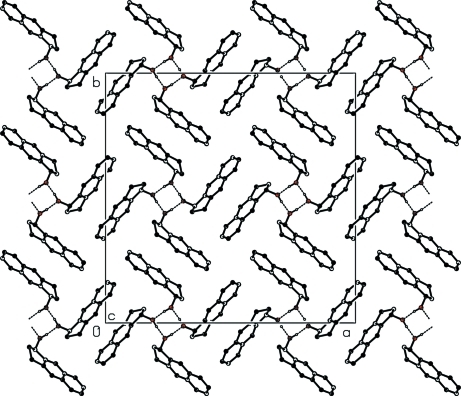
The packing and hydrogen bonding of the title compound, viewing down the c axis. H atoms not involved in hydrogen bonding have been omitted.

## supplementary crystallographic information

### Comment

The treatment of benz[*f*]indanone with sodium borohydride in THF/MeOH for 2 h afforded the corresponding benz[*f*]indan-1-ol in an 89% yield (Carpino & Lin, 1990). Two signal groups, aliphatic and aromatic were observed in ^1^H-NMR spectra. The H2 and H3 protons exhibited a AA'BB' splitting pattern. H1 appeared at lower magnetic field than the 2,3 H atoms. This is due to the electronegativity of oxygen atom attached to C-1. The signal observed at δ 5.39 as a broad singlet could be attributed to OH group. Moreover a thirteen line ^13^C-NMR spectrum supports the proposed structure. The exact configuration of the molecule was established by X-ray diffraction.

The molecular structure of the title compound (I) is shown in Fig. 1. All bond lengths and angles in the title compound (I) show normal values (Allen *et al.*, 1987; Çelik *et al.*, 2009). The cyclopentene ring (C8–C9/C11–C13) fused with the naphthalene group (C1–C10) adopts an envelope conformation [puckering parameters: Q(2) = 0.2503 (18) Å, φ(2) = 110.1 (4)°; (Cremer & Pople, 1975)] with atom C12 deviating from the ring plane. The naphthalene group is essentially planar with a maximum deviation of -0.013 (1) Å for C8. The torsion angles C9–C8–C13–O1 and C7–C8– C13–O1 are -141.52 (12) and 38.33 (19) °, respectively.

In the crystal, neighbouring molecules are linked with intermolecular O—H···O hydrogen bonds, forming *R*_4_^4^(8) ring motifs (Bernstein *et al.*, 1995). A weak π -π stacking interaction [*Cg*3···*Cg*3^iii^ = 3.8981 (8) Å (symmetry code: (iii) = -*x*, -*y*, -*z*)] between the C5–C10 benzene rings and two C—H···π interactions contribute to the stabilization of the crystal packing.

### Experimental

To an ice-cold, stirred solution of benz[*f*]indanone (4.0 g, 22 mmol) in THF/MeOH (30/20 ml) mixture was added NaBH_4_ (0.34 g, 8.8 mmol). The reaction mixture was allowed to warm to room temperature. After the completion of the reaction, 2 h, water was added to the reaction mixture which was extracted with diethyl ether (3×50 ml), dried over MgSO_4_ and concentrated in *vacuo* affording benz[*f*]indan-1-ol which was crystallized from hexane/chloroform as yellow needles (3.6 g, 89%). ^1^H-NMR (400 MHz, CDCl_3_): δ 2.0–2.09 (m, 2H, H2), 2.54–2.60 (m, 1H, H3), 2.94–3.02 (m, 1H, H3'), 3.18–3.26 (m, 1H, H1), 5.39 (brs, 1H, OH), 7.43–7.49 (m, 2H, ArH), 7.70 (brs, 1H, ArH), 7.80–7.85 (brs, 2H, ArH), 7.87 (brs, 1H, ArH); ^13^C-NMR (100 MHz, CDCl_3_): δ 29.3, 36.6, 75.9, 122.7, 122.9, 125.2, 125.8, 127.6, 128.2, 132.9, 133.9, 141.5, 144.3.

### Refinement

The H atom of the hydroxyl group was located in a difference Fourier map and refined freely. Carbon-bound H-atoms were placed in calculated positions and refined with constrained C—H bond lengths of 0.93 Å for aromatic, 0.97 Å for methylene and 0.98 Å for methine H atoms, and *U*_iso_(H) = 1.2 *U*_eq_(C) allowing them to ride on the parent C atom. The (6 6 0), (-6 12 0) and (-2 6 0) reflections were omitted owing to bad disagreement.

### Crystal data

**Table:**

C_13_H_12_O	*D*_x_ = 1.253 Mg m^−^^3^
*M**_r_* = 184.23	Cu *K*α radiation, λ = 1.5418 Å
Tetragonal, *I*4_1_/*a*	Cell parameters from 2801 reflections
Hall symbol: -I 4ad	θ = 3.5–68.0°
*a* = 25.3105 (4) Å	µ = 0.61 mm^−^^1^
*c* = 6.0995 (2) Å	*T* = 299 K
*V* = 3907.47 (18) Å^3^	Needle, colourless
*Z* = 16	0.46 × 0.17 × 0.11 mm
*F*(000) = 1568	

### Data collection

**Table:**

Agilent Xcalibur Ruby Gemini diffractometer	1791 independent reflections
Radiation source: Enhance (Cu) X-ray Source	1580 reflections with *I* > 2σ(*I*)
Graphite monochromator	*R*_int_ = 0.026
Detector resolution: 10.2673 pixels mm^-1^	θ_max_ = 68.2°, θ_min_ = 3.5°
ω scans	*h* = −30→18
Absorption correction: multi-scan (*CrysAlis PRO*; Agilent, 2011)	*k* = −25→28
*T*_min_ = 0.884, *T*_max_ = 0.936	*l* = −7→6
5702 measured reflections	

### Refinement

**Table:**

Refinement on *F*^2^	Primary atom site location: structure-invariant direct methods
Least-squares matrix: full	Secondary atom site location: difference Fourier map
*R*[*F*^2^ > 2σ(*F*^2^)] = 0.044	Hydrogen site location: inferred from neighbouring sites
*wR*(*F*^2^) = 0.126	H atoms treated by a mixture of independent and constrained refinement
*S* = 1.04	*w* = 1/[σ^2^(*F*_o_^2^) + (0.0709*P*)^2^ + 0.8441*P*] where *P* = (*F*_o_^2^ + 2*F*_c_^2^)/3
1791 reflections	(Δ/σ)_max_ < 0.001
131 parameters	Δρ_max_ = 0.10 e Å^−^^3^
0 restraints	Δρ_min_ = −0.17 e Å^−^^3^

### Special details

**Table:**

Geometry. Bond distances, angles *etc*. have been calculated using the rounded fractional coordinates. All su's are estimated from the variances of the (full) variance-covariance matrix. The cell e.s.d.'s are taken into account in the estimation of distances, angles and torsion angles
Refinement. Refinement on *F*^2^ for ALL reflections except those flagged by the user for potential systematic errors. Weighted *R*-factors *wR* and all goodnesses of fit *S* are based on *F*^2^, conventional *R*-factors *R* are based on *F*, with *F* set to zero for negative *F*^2^. The observed criterion of *F*^2^ > σ(*F*^2^) is used only for calculating -*R*-factor-obs *etc*. and is not relevant to the choice of reflections for refinement. *R*-factors based on *F*^2^ are statistically about twice as large as those based on *F*, and *R*-factors based on ALL data will be even larger.

### Fractional atomic coordinates and isotropic or equivalent isotropic displacement parameters (Å^2^)

**Table:**

	*x*	*y*	*z*	*U*_iso_*/*U*_eq_	
O1	0.18860 (4)	0.01236 (4)	0.19317 (19)	0.0630 (4)	
C1	0.03049 (6)	−0.12900 (6)	0.2324 (3)	0.0625 (5)	
C2	−0.00864 (7)	−0.16213 (7)	0.1633 (3)	0.0760 (6)	
C3	−0.03313 (7)	−0.15412 (8)	−0.0393 (4)	0.0800 (7)	
C4	−0.01801 (6)	−0.11348 (7)	−0.1700 (3)	0.0717 (6)	
C5	0.02266 (5)	−0.07803 (6)	−0.1061 (2)	0.0550 (4)	
C6	0.04727 (5)	−0.08598 (5)	0.1014 (2)	0.0505 (4)	
C7	0.08716 (5)	−0.05063 (5)	0.1706 (2)	0.0494 (4)	
C8	0.10205 (5)	−0.00984 (5)	0.0392 (2)	0.0487 (4)	
C9	0.07840 (6)	−0.00232 (5)	−0.1682 (2)	0.0566 (4)	
C10	0.03969 (6)	−0.03549 (6)	−0.2388 (2)	0.0615 (5)	
C11	0.10340 (8)	0.04480 (6)	−0.2785 (3)	0.0755 (6)	
C12	0.15329 (8)	0.05519 (7)	−0.1448 (3)	0.0794 (6)	
C13	0.14311 (6)	0.03180 (5)	0.0832 (2)	0.0570 (4)	
H1	0.04640	−0.13480	0.36780	0.0750*	
H1O	0.2035 (8)	−0.0143 (8)	0.108 (3)	0.084 (6)*	
H2	−0.01910	−0.19020	0.25160	0.0910*	
H3	−0.05990	−0.17680	−0.08470	0.0960*	
H4	−0.03470	−0.10870	−0.30450	0.0860*	
H7	0.10330	−0.05530	0.30630	0.0590*	
H10	0.02430	−0.03020	−0.37550	0.0740*	
H11A	0.07990	0.07510	−0.27460	0.0910*	
H11B	0.11210	0.03690	−0.42990	0.0910*	
H12A	0.16010	0.09280	−0.13440	0.0950*	
H12B	0.18360	0.03830	−0.21260	0.0950*	
H13	0.12710	0.05930	0.17480	0.0680*	

### Atomic displacement parameters (Å^2^)

**Table:**

	*U*^11^	*U*^22^	*U*^33^	*U*^12^	*U*^13^	*U*^23^
O1	0.0583 (6)	0.0621 (6)	0.0686 (7)	−0.0038 (4)	−0.0021 (5)	−0.0055 (5)
C1	0.0597 (8)	0.0665 (8)	0.0614 (9)	−0.0024 (6)	0.0036 (7)	0.0065 (7)
C2	0.0656 (9)	0.0701 (9)	0.0924 (13)	−0.0087 (7)	0.0122 (9)	−0.0013 (9)
C3	0.0583 (9)	0.0830 (11)	0.0987 (14)	−0.0093 (8)	0.0023 (9)	−0.0230 (10)
C4	0.0569 (8)	0.0917 (11)	0.0666 (10)	0.0124 (7)	−0.0110 (7)	−0.0251 (9)
C5	0.0505 (7)	0.0668 (8)	0.0476 (7)	0.0136 (6)	−0.0029 (5)	−0.0088 (6)
C6	0.0464 (6)	0.0588 (7)	0.0464 (7)	0.0077 (5)	0.0019 (5)	0.0000 (5)
C7	0.0496 (6)	0.0603 (7)	0.0384 (6)	0.0049 (5)	−0.0036 (5)	0.0053 (5)
C8	0.0510 (7)	0.0548 (7)	0.0402 (6)	0.0094 (5)	0.0042 (5)	0.0032 (5)
C9	0.0703 (8)	0.0599 (8)	0.0396 (7)	0.0203 (6)	0.0043 (6)	0.0058 (6)
C10	0.0687 (9)	0.0767 (9)	0.0391 (7)	0.0237 (7)	−0.0097 (6)	−0.0025 (6)
C11	0.1115 (14)	0.0666 (9)	0.0485 (8)	0.0158 (9)	0.0098 (8)	0.0158 (7)
C12	0.0947 (12)	0.0727 (10)	0.0707 (11)	−0.0038 (9)	0.0191 (9)	0.0210 (8)
C13	0.0636 (8)	0.0532 (7)	0.0541 (8)	0.0046 (6)	0.0085 (6)	0.0034 (6)

### Geometric parameters (Å, º)

**Table:**

O1—C13	1.4205 (18)	C9—C11	1.508 (2)
O1—H1O	0.93 (2)	C11—C12	1.526 (3)
C1—C6	1.416 (2)	C12—C13	1.533 (2)
C1—C2	1.364 (2)	C1—H1	0.9300
C2—C3	1.397 (3)	C2—H2	0.9300
C3—C4	1.357 (3)	C3—H3	0.9300
C4—C5	1.420 (2)	C4—H4	0.9300
C5—C10	1.414 (2)	C7—H7	0.9300
C5—C6	1.4249 (17)	C10—H10	0.9300
C6—C7	1.4135 (18)	C11—H11A	0.9700
C7—C8	1.3603 (18)	C11—H11B	0.9700
C8—C13	1.5043 (19)	C12—H12A	0.9700
C8—C9	1.4124 (18)	C12—H12B	0.9700
C9—C10	1.360 (2)	C13—H13	0.9800
			
C13—O1—H1O	108.4 (12)	C6—C1—H1	120.00
C2—C1—C6	121.07 (16)	C1—C2—H2	120.00
C1—C2—C3	120.40 (17)	C3—C2—H2	120.00
C2—C3—C4	120.31 (17)	C2—C3—H3	120.00
C3—C4—C5	121.49 (16)	C4—C3—H3	120.00
C4—C5—C6	118.13 (13)	C3—C4—H4	119.00
C6—C5—C10	118.86 (12)	C5—C4—H4	119.00
C4—C5—C10	123.02 (13)	C6—C7—H7	120.00
C1—C6—C5	118.61 (12)	C8—C7—H7	120.00
C5—C6—C7	119.22 (12)	C5—C10—H10	120.00
C1—C6—C7	122.18 (12)	C9—C10—H10	120.00
C6—C7—C8	120.16 (11)	C9—C11—H11A	111.00
C7—C8—C9	120.84 (12)	C9—C11—H11B	111.00
C7—C8—C13	128.17 (11)	C12—C11—H11A	111.00
C9—C8—C13	110.99 (11)	C12—C11—H11B	111.00
C8—C9—C10	120.37 (12)	H11A—C11—H11B	109.00
C8—C9—C11	109.16 (13)	C11—C12—H12A	111.00
C10—C9—C11	130.46 (13)	C11—C12—H12B	111.00
C5—C10—C9	120.55 (12)	C13—C12—H12A	110.00
C9—C11—C12	104.18 (14)	C13—C12—H12B	110.00
C11—C12—C13	106.21 (15)	H12A—C12—H12B	109.00
O1—C13—C12	115.20 (13)	O1—C13—H13	108.00
C8—C13—C12	102.99 (11)	C8—C13—H13	108.00
O1—C13—C8	113.68 (10)	C12—C13—H13	108.00
C2—C1—H1	119.00		
			
C6—C1—C2—C3	−0.1 (3)	C6—C7—C8—C13	179.66 (12)
C2—C1—C6—C5	−0.4 (2)	C7—C8—C9—C10	0.9 (2)
C2—C1—C6—C7	179.04 (14)	C7—C8—C9—C11	−178.70 (13)
C1—C2—C3—C4	0.4 (3)	C13—C8—C9—C10	−179.25 (13)
C2—C3—C4—C5	−0.1 (3)	C13—C8—C9—C11	1.16 (16)
C3—C4—C5—C6	−0.4 (2)	C7—C8—C13—O1	38.33 (19)
C3—C4—C5—C10	179.53 (16)	C7—C8—C13—C12	163.67 (14)
C4—C5—C6—C1	0.7 (2)	C9—C8—C13—O1	−141.52 (12)
C4—C5—C6—C7	−178.80 (13)	C9—C8—C13—C12	−16.18 (15)
C10—C5—C6—C1	−179.26 (13)	C8—C9—C10—C5	−0.2 (2)
C10—C5—C6—C7	1.25 (19)	C11—C9—C10—C5	179.32 (15)
C4—C5—C10—C9	179.17 (14)	C8—C9—C11—C12	14.52 (17)
C6—C5—C10—C9	−0.9 (2)	C10—C9—C11—C12	−165.02 (16)
C1—C6—C7—C8	179.97 (13)	C9—C11—C12—C13	−24.27 (17)
C5—C6—C7—C8	−0.57 (19)	C11—C12—C13—O1	148.97 (12)
C6—C7—C8—C9	−0.50 (19)	C11—C12—C13—C8	24.64 (16)

### Hydrogen-bond geometry (Å, º)

Cg2 is a centroid of the C1–C6 benzene ring.

**Table:**

*D*—H···*A*	*D*—H	H···*A*	*D*···*A*	*D*—H···*A*
O1—H1*O*···O1^i^	0.93 (2)	1.78 (2)	2.7113 (15)	174.4 (18)
C3—H3···*Cg*2^ii^	0.93	2.71	3.633 (2)	171
C11—H11*A*···*Cg*2^iii^	0.97	2.89	3.706 (2)	142

Symmetry codes: (i) −*y*+1/4, *x*−1/4, *z*−1/4; (ii) −*y*−1/4, *x*−1/4, −*z*−1/4; (iii) −*x*, −*y*, −*z*.
